# Cross-sectional study of major procedure codes among hospitalized patients with traumatic brain injury by level of injury severity in the 2004 to 2014 Nationwide Inpatient Sample

**DOI:** 10.1097/MD.0000000000024438

**Published:** 2021-02-12

**Authors:** Hind A. Beydoun, Catherine Butt, May A. Beydoun, Sharmin Hossain, Shaker M. Eid, Alan B. Zonderman

**Affiliations:** a Department of Research Programs, Fort Belvoir Community Hospital; b Intrepid Spirit Center, Defense and Veterans Brain Injury Center, Fort Belvoir, VA; c Laboratory of Epidemiology and Population Sciences, National Institute on Aging, NIA/NIH/IRP; d Department of Medicine, Johns Hopkins School of Medicine, Baltimore, MD.

**Keywords:** hospital, injury, procedure, traumatic brain injury

## Abstract

Despite its public health significance, TBI management across US healthcare institutions and patient characteristics with an emphasis on utilization and outcomes of TBI-specific procedures have not been evaluated at the national level.

We aimed to characterize top 10 procedure codes among hospitalized adults with TBI as primary diagnosis by injury severity.

A Cross-sectional study was conducted using 546, 548 hospitalization records from the 2004 to 2014 Nationwide Inpatient Sample were analyzed.

Data elements of interest included injury, patient, hospital characteristics, procedures, in-hospital death and length of stay.

Ten top procedure codes were “Closure of skin and subcutaneous tissue of other sites”, “Insertion of endotracheal tube”, “Continuous invasive mechanical ventilation for less than 96 consecutive hours”, “Venous catheterization (not elsewhere classified)”, “Continuous invasive mechanical ventilation for 96 consecutive hours or more”, “Transfusion of packed cells”, “Incision of cerebral meninges”, “Serum transfusion (not elsewhere classified)”, “Temporary tracheostomy”, and “Arterial catherization”. Prevalence rates ranged between 3.1% and 15.5%, with variations according to injury severity and over time. Whereas “Closure of skin and subcutaneous tissue of other sites” was associated with fewer in-hospital deaths and shorter hospitalizations, “Temporary tracheostomy” was associated with fewer in-hospital deaths among moderate-to-severe TBI patients, and “Continuous invasive mechanical ventilation for less than 96 consecutive hours” was associated with shorter hospitalizations among severe TBI patients. Other procedures were associated with worse outcomes.

Nationwide, the most frequently reported hospitalization procedure codes among TBI patients aimed at homeostatic stabilization and differed in prevalence, trends, and outcomes according to injury severity.

## Abstract 2

Supplemental Digital Content is available in the text

## 1 Introduction

Traumatic brain injury (TBI) remains a leading cause of morbidity and mortality worldwide.^[1–3]^ TBIs are often classified into mild, moderate, and severe subtypes on the basis of Glasgow Coma Scale (GCS) scores, duration of loss of consciousness (LOC), and duration of posttraumatic amnesia (PTA).^[4]^ The Centers for Disease Control and Prevention (CDC) estimates that, each year, mild, moderate, and severe TBI affect 1.7 million people in the United States (US), of whom 275,000 are hospitalized, 90,000 are left with significant disability and/or physical impairment and 52,000 are deceased.^[2,5,6]^

TBI represents a major contributor to premature mortality, is a major consumer of healthcare services and may place a substantial burden on families and caregivers.^[1,7,8]^ Whereas nearly 40% of US deaths from acute injury have been attributed to moderate-to-severe TBI,^[9,10]^ TBI neuropsychological sequelae can range from short-term memory and neurocognitive deficits to long-term physical, psychological, emotional, and social difficulties, and can negatively impact a TBI survivors’ quality of life, potentially leading to loss of productivity for 6 to 12 months or longer.^[1,5,11,12]^

Due to its heterogeneous nature, TBI diagnosis and treatment can be challenging.^[13]^ In 2011, a congressional report was published by the Department of Defense that summarizes the effectiveness of multiple neuroimaging modalities at evaluating the TBI spectrum from concussion to coma.^[14]^ However, it remains unclear whether any of these modalities is sufficient for diagnosing TBI. Despite clinical trials focused on neuroprotective strategies, pharmacologic, and surgical approaches,^[13]^ there is no single treatment option for a complex condition such as TBI. Although a primary injury may result in damage, secondary injuries resulting from inflammatory reactions are likely to determine prognosis after moderate-to-severe TBI.^[15,16]^ Therefore, acute management of TBI has focused on prevention of secondary injuries caused by hypotension and hypoxia, as well as prevention of venous thromboembolism, stress ulcer, and seizures, while maintaining cerebral perfusion pressure and optimizing nutritional and metabolic status.^[17]^ Finally, postacute rehabilitation services have been linked to better functioning among TBI patients.^[1]^

Although the burden of TBI has been established at the national level, that of resource use among patients with TBI remains elusive. Despite its public health significance, patterns of TBI management using specific diagnostic and treatment modalities have not been evaluated among US hospitalized patients. This information can aid healthcare organizations when planning for future resource allocation. Accordingly, the purpose of this exploratory cross-sectional study was to analyze data from the 2004 to 2014 Nationwide Inpatient Sample (NIS) to identify and characterize utilization and outcomes of the top 10 diagnostic and treatment procedure codes among hospitalized adults with a primary diagnosis of TBI, and to examine disparities in the diagnosis and treatment of TBI by injury severity. Given the acute nature of the hospital setting, we expect to identify distinct patterns of resource utilization according to TBI severity, with the majority of patients undergoing procedures aimed at homeostatic stabilization as well as prevention of secondary injuries post-TBI.

## 2 Methods

### 2.1 Data source

Secondary analyses of existing data from the Agency for Healthcare Research and Quality (AHRQ) Healthcare Cost and Utilization Project (HCUP) NIS were performed. The NIS is the largest publicly available, all-payer inpatient care database of community (non-federal) hospitals in the US. It consists of 5 to 8 million hospital discharge records sampled from thousands of hospitals on an annual basis since 1988. Each year, a 20% stratified probability sample of hospitals (before 2012) or hospital discharge records (since 2012) is selected from all participating HCUP US states, based on multiple hospital characteristics namely ownership/control, bed size, teaching status, urban/rural location, and US region. The NIS data elements include patient demographics, at least 15 diagnoses and 15 procedures, hospital course and outcomes, with no protected health information. The original research project as designed by AHRQ was approved by an Institutional Review Board in accordance with principles outlined by the Declaration of Helsinki, and this project received a determination of research not involving human subjects since it involved secondary analysis of publicly available de-identified data. As such, informed consent was deemed not necessary for this research project.

### 2.2 Eligibility criteria

Inclusion and exclusion criteria were defined based on previously published TBI studies that have used the NIS or other national databases.^[5,7,10,18–21]^ The study population consists of hospitalization records from the 2004 to 2014 NIS databases that met the following inclusion criteria:

1. Age ≥ 18 years;
2. Clinical Classifications Software (CCS) code of 233 assigned by AHRQ for TBI;
3. Primary diagnosis of TBI using ICD-9-CM codes recommended by the CDC, whereby variable DX1 was coded as “fracture of cranial vault, skull base, or facial bone with intracranial injury” (800.0–801.9, 803.0–804.9) or “concussion, cerebral contusion, subdural hematoma, epidural hematoma, other, and unspecified traumatic intracranial hemorrhage” (850–854.19), “injury to the optic nerve and pathways” (950.1–950.3) and/or “head injury, unspecified” (959.01);
4. at least one of the first 15 procedure codes is non-missing.

Hospitalization records were not considered eligible if: primary diagnosis was for TBI history (V1552); elective hospital admission; Abbreviated Injury Severity (AIS) score was deemed non-survivable; missing data on key patient and hospital characteristics. Patients who died within 48 hours of hospital admission were not excluded because in-hospital death was evaluated as a healthcare utilization outcome.^[20]^

### 2.3 Patient and hospital characteristics

Patient characteristics were defined as age, race/ethnicity, primary payer, weekend admission as well as year, and quarter of admission. Hospital characteristics were defined as region, control, location, teaching status, and bed size.

### 2.4 Injury severity

In the absence of data elements for GCS, LOC, and PTA, injury severity among TBI-affected patients was calculated using AIS. Using a Stata program, ICD-9-CM diagnostic codes were translated into AIS scores specific to the head and/or neck region. The highest AIS score was selected to categorize injury severity as ranging from 1 (“minor”) to 6 (“unsurvivable”), and records with AIS of 6 were excluded. Subsequently, “mild” TBI was defined among patients with AIS score between 1 and 2, “moderate” TBI among patients with AIS score of 3 and “severe” TBI among patients with AIS between 4 and 5, as previously described.^[1,5]^

### 2.5 Procedure codes

Using procedure data elements, the original database was transposed to identify the most frequent diagnostic and/or treatment procedure codes. Subsequently, top 10 procedure codes with frequency ≥10,000 were selected for further analyses irrespectively of their assumed relatedness to TBI (Appendix 1, http://links.lww.com/MD/F658). An indicator variable was created for each procedure to flag hospital records that utilized these procedures.

### 2.6 Healthcare utilization outcomes

Major procedures were examined in relation to in-hospital death and length of hospital stay. Because of skewed distributions, length of stay was log_e_-transformed prior to regression modeling.

### 2.7 Statistical analysis

All statistical analyses were conducted using Stata release 15 (StataCorp, College Station, TX), taking complex sampling design into consideration. Descriptive statistics included mean (± standard error) and frequencies with percentages, as appropriate. Bivariate comparisons were evaluated using Chi-Squared test, design-based F-tests, regression modeling to evaluate trends by injury severity and/or year of admission. Linear and logistic regression models were constructed to estimate crude or adjusted β coefficients and odds ratios (cOR and aOR) with their 95% confidence intervals (CI). First, we examined disparities in patient and hospital characteristics as well as major procedures among mild, moderate and severe TBI. Second, we examined trends in major procedures over time, and according to injury severity. Third, we examined the relationship between major procedures and selected in-hospital outcomes, before and after adjustment for patient and hospital characteristics. Complete subject analyses were performed based on available sub-samples for variables under evaluation. Two-sided statistical tests were conducted with *P* < .05 considered statistically significant.

### 2.8 Data availability statement

The authors have access to the de-identified HCUP NIS raw data through a data-use agreement with AHRQ. Therefore, these raw data are restricted and cannot be publicly shared, for legal or ethical reasons.

## 3 Results

### 3.1 Descriptive statistics

**Figure 1:**
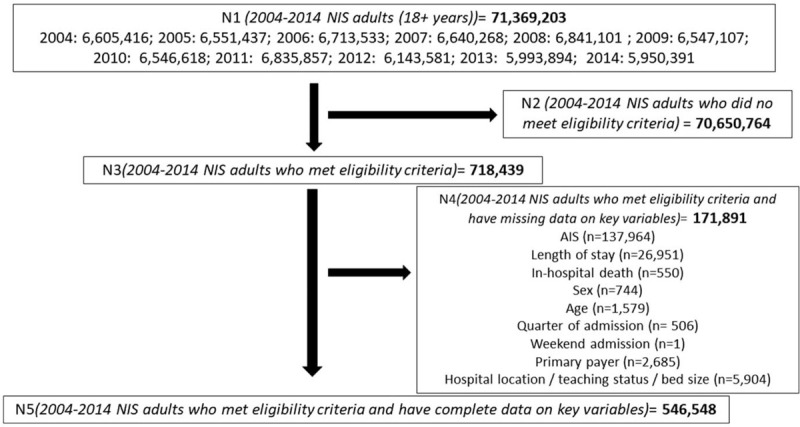
Study flowchart -- NIS 2004 to 2014.

A total of 71,369,203 hospitalization records from the 2004 to 2014 NIS databases corresponded to adult patients, 18 years and older. Of those, 718,439 met all pre-specified eligibility criteria except for non-missing data on key variables. After exclusion of patients with missing data on key characteristics, 546, 548 hospital discharge records (168,089 “mild” TBI, 182,210 “moderate” TBI, and 196,249 “severe” TBI) were available for subsequent analyses (Fig. 1).

Table 1 presents baseline characteristics by injury severity. Nearly 30.8% of hospitalization records corresponded to “mild” TBI, 33.3% to “moderate’ TBI and 35.9% to “severe’ TBI. Approximately 61% of hospitalization records consisted of male patients, 44% to elderly patients, 48% of Whites, 69% of patients who sought care on weekdays, and 31% of patients with private insurance. Furthermore, 36% of records corresponded to patients admitted at Southern hospitals, 54% to those admitted to private/governmental hospitals, 67% to patients admitted to urban/teaching hospitals, and 73% to patients admitted to large bed-sized hospitals. The distribution of records by these characteristics differed significantly according to injury severity.

**Table 1:** Characteristics of patients with traumatic brain injury by level of injury severity, Nationwide Inpatient Sample, 2004 to 2014.

	Injury Severity Level (%)			Total (%)
	Mild (n = 168,089)	Moderate (n = 182,210)	Severe (n = 196,249)	n = 546,548
Sex:	*P* < .0001			
Male	60.4	65.2	58.5	60.9
Female	39.6	35.8	41.4	39.0
Age (years):	*P* < .0001			
18–24	15.1	12.4	5.6	10.8
25–29	7.8	6.6	2.9	5.7
30–34	7.2	6.4	2.9	5.4
35–39	6.4	5.2	2.5	4.6
40–44	6.9	5.9	3.2	5.3
45–49	7.7	6.9	4.1	6.1
50–54	7.6	7.5	5.5	6.8
55–59	6.3	6.6	5.5	6.1
60–64	5.4	5.9	5.9	5.7
65+	29.6	36.6	61.9	43.5
Race / Ethnicity:	*P* < .0001			
White	47.5	48.3	48.3	48.1
Black	6.9	5.8	5.8	6.1
Hispanic	7.7	7.8	5.8	7.1
Other	4.3	4.5	4.4	4.4
Unknown	33.7	33.6	35.7	34.4
Year of admission:	*P* < .0001			
2004	9.8	8.5	7.0	8.4
2005	8.8	7.2	6.8	7.5
2006	10.4	8.7	7.9	8.9
2007	9.0	9.1	8.0	8.7
2008	9.7	9.2	8.5	9.1
2009	9.3	11.3	8.9	9.1
2010	10.7	9.0	10.9	10.9
2011	8.3	9.0	9.8	9.1
2012	8.6	9.6	10.4	9.6
2013	7.8	9.2	10.6	9.3
2014	7.5	9.1	11.1	9.3
Admission quarter:	*P* < .0001			
1st quarter	22.9	22.5	23.6	23.0
2nd quarter	25.9	25.1	24.5	25.1
3rd quarter	26.6	26.9	25.9	26.5
4th quarter	24.6	25.5	26.0	25.4
Weekend admission status:	*P* < .0001			
Monday–Friday	67.6	67.1	72.3	69.1
Saturday–Sunday	32.4	32.8	27.7	30.9
Primary payer:	*P* < .0001			
Medicare	28.4	34.5	58.3	41.2
Medicaid	9.8	10.3	7.5	9.2
Private insurance	38.9	33.3	21.8	30.9
Self-Pay	13.3	12.8	6.9	10.8
No charge	8.7	9.9	5.7	8.0
Other	8.8	8.2	4.9	7.2
Hospital region:	*P* < .0001			
Northeast	21.8	18.7	19.2	19.8
Midwest	24.2	21.4	22.2	22.6
South	32.7	38.5	37.6	36.4
West	21.4	21.5	20.9	21.2
Hospital control:	*P* < .0001			
Government or Private	55.3	56.6	50.5	54.0
Government, non-federal	4.9	4.1	3.9	4.3
Private, not-for-profit	16.3	15.2	17.5	16.4
Private, investor-owned	5.6	4.8	5.3	5.2
Private	2.5	0.9	1.1	1.5
Unknown	15.3	18.3	21.7	18.6
Location and teaching status:	*P* < .0001			
Rural	9.2	3.5	4.4	5.6
Urban – Non-Teaching	29.6	24.5	29.1	27.7
Urban – Teaching	61.2	71.9	66.5	66.8
Hospital bed size:	*P* < .0001			
Small	8.0	4.9	6.4	6.4
Medium	22.6	19.8	20.5	20.9
Large	69.4	75.3	73.1	72.7

We identified 10 major procedure codes which were labeled from most to least frequent, as “Closure of skin and subcutaneous tissue of other sites”, “Insertion of endotracheal tube”, “Continuous invasive mechanical ventilation for less than 96 consecutive hours”, “Venous catheterization (not elsewhere classified)”, “Continuous invasive mechanical ventilation for 96 consecutive hours or more”, “Transfusion of packed cells”, “Incision of cerebral meninges”, “Serum transfusion (not elsewhere classified)”, “Temporary tracheostomy”, and “Arterial catherization”. The prevalence rates of these procedure codes ranged between 3.1% and 15.5% in the overall sample.

### 3.2 Key findings

As shown in Table 2, there were statistically significant differences in the prevalence rates of all major procedures according to injury severity. Specifically, there were increasing trends in the prevalence of several procedure codes (insertion of endotracheal tube, transfusion of packed cells, incision of cerebral meninges, serum transfusion (not elsewhere classified)) with increasing injury severity. By contrast, a decreasing trend in prevalence of closure of skin and subcutaneous tissue of other sites was observed with increasing injury severity. Other procedure codes did not exhibit an increasing or decreasing trend by injury severity. Supplemental Table 1, http://links.lww.com/MD/F659 presents the prevalence of major procedures by year of admission and injury severity. Although no clear trends were observed, with few exceptions, procedure rates differed by year of admission as well as injury severity.

**Table 2:** Major diagnostic and/or treatment procedures by level of injury severity, Nationwide Inpatient Sample, 2004 to 2014.

	Injury severity level (%)			Total (%)
	Mild (n = 168,089)	Moderate (n = 182,210)	Severe (n = 196,249)	n = 546,548
Procedure #1: 8659 Closure of skin and subcutaneous tissue of other sites	*P* < .0001			
% yes	19.9	17.0	10.3	15.5
Procedure #2: 9604 Insertion of endotracheal tube	*P* < .0001			
% yes	6.7	14.5	12.2	11.3
Procedure #3: 9671 Continuous invasive mechanical ventilation for less than 96 consecutive hours	*P* < .0001			
% yes	7.4	12.6	10.9	10.4
Procedure #4: 3893 Venous catheterization (not elsewhere classified)	*P* < .0001			
% yes	3.5	10.9	9.9	8.3
Procedure #5: 9672 Continuous invasive mechanical ventilation for 96 consecutive hours or more	*P* < .0001			
% yes	2.1	11.0	8.2	7.3
Procedure #6: 9904 Transfusion of packed cells	*P* <.0001			
% yes	4.9	7.7	8.5	7.2
Procedure #7: 0131 Incision of cerebral meninges	*P* < .0001			
% yes	0.2	3.5	15.2	6.7
Procedure #8: 9907 Serum transfusion (not elsewhere classified)	*P* < .0001			
% yes	0.9	4.0	6.8	4.1
Procedure #9: 311 Temporary tracheostomy	*P* < .0001			
% yes	1.1	6.6	4.3	4.1
Procedure #10: 3891 Arterial catherization	*P* < .0001			
% yes	0.9	4.5	3.5	3.1

Table 3 presents major procedure codes as predictors of in-hospital outcomes (death and length of stay) among TBI patients by injury severity. In-hospital death rate was 6.5% (0.9% among “mild” TBI, 7.2% among “moderate” TBI, 10.7% among “severe” TBI, *P* < .0001). Similarly, mean length of hospital stay was 6.9 days (4.7 days among “mild” TBI, 8.2 days among “moderate” TBI, 7.8 days among “severe” TBI). Whereas “Closure of skin and subcutaneous tissue of other sites” was associated with fewer in-hospital deaths and shorter hospitalizations, “Temporary tracheostomy” was associated with fewer in-hospital deaths among moderate-to-severe TBI patients, and “Continuous invasive mechanical ventilation for less than 96 consecutive hours” was associated with shorter hospitalizations among severe TBI patients. Other procedures were associated with worse outcomes. With few exceptions, the relationship between these top 10 procedure codes and in-hospital outcomes, differed significantly according to injury severity.

**Table 3:** Major diagnostic and treatment procedures as predictors of in-hospital outcomes of patients with traumatic brain injury by level of injury severity, Nationwide Inpatient Sample, 2004 to 2014.

	In-hospital death		Log_e_-length of stay	
Overall:	OR (95% CI)		β (95% CI)	
	Unadjusted	Adjusted^∗^	Unadjusted	Adjusted^∗^
* Procedure #1*	.55 (.53, .57)	.60 (.58, .62)	−.092 (−.099, −.085)	−.065 (−.072, −.058)
* Procedure #2*	7.92 (7.74, 8.11)	9.30 (9.07, 9.53)	.78 (.77, .79)	.77 (.76, .78)
* Procedure #3*	9.22 (9.00, 9.43)	11.84 (11.53, 12.14)	.14 (.13, .15)	.14 (.13, .15)
* Procedure #4*	6.11 (5.96, 6.27)	6.38 (6.22, 6.55)	1.07 (1.06, 1.08)	1.04 (1.03, 1.05)
* Procedure #5*	5.00 (4.87, 5.14)	5.45 (5.34, 5.65)	1.67 (1.66, 1.68)	1.66 (1.65, 1.66)
* Procedure #6*	4.19 (4.08, 4.31)	4.07 (3.95, 4.18)	.83 (.82, .84)	0.79 (0.79, 0.81)
* Procedure #7*	2.74 (2.66, 2.83)	2.26 (2.19, 2.34)	.78 (.77, .79)	0.71 (0.70, 0.72)
* Procedure #8*	5.46 (5.28, 5.64)	4.72 (4.56, 4.88)	.42 (.41, .43)	0.37 (0.36, 0.38)
* Procedure #9*	1.16 (1.10, 1.22)	1.19 (1.13, 1.26)	1.96 (1.95, 1.97)	1.94 (1.93, 1.95)
* Procedure #10*	7.97 (7.70, 8.25)	8.86 (8.54, 9.20)	.84 (.82, .85)	0.81 (0.79, 0.83)
* *(%) or Mean ± SEM	(6.5%)		6.97 ± .01	
MILD:				
* Procedure #1*	.62 (.54, .72)	.66 (.57, .77)	−.02 (−.03, −0.01)	−.009 (−.02, .001)
* Procedure #2*	11.3 (10.2, 12.5)	18.4 (16.3, 20.7)	.53 (.54, .58)	.59 (.57, .61)
* Procedure #3*	6.5 (5.8, 7.2)	12.3 (10.9, 13.9)	.22 (.19, .23)	.26 (.24, .27)
* Procedure #4*	13.2 (11.9, 14.8)	13.4 (11.9, 15.1)	1.27 (1.25, 1.29)	1.23 (1.21, 1.26)
* Procedure #5*	14.9 (13.2, 16.9)	15.7 (13.7, 17.9)	1.94 (1.92, 1.96)	1.89 (1.87, 1.91)
* Procedure #6*	5.9 (5.3, 6.7)	4.9 (4.3, 5.5)	1.03 (1.00, 1.04)	.97 (.96, .99)
* Procedure #7*	19.6 (13.9, 27.4)	13.1 (9.0, 19.0)	1.22 (1.11, 1.34)	1.14 (1.03, 1.26)
* Procedure #8*	13.2 (11.2, 15.8)	9.4 (7.8, 11.4)	.85 (.79, .90)	.78 (.73, .83)
* Procedure #9*	5.7 (4.6, 7.1)	5.5 (4.4, 6.9)	2.18 (2.16, 2.22)	2.12 (2.09, 2.15)
* Procedure #10*	17.5 (14.9, 20.4)	19.5 (16.4, 23.1)	1.18 (1.13, 1.23)	1.14 (1.09, 1.19)
(%) or Mean ± SEM	(0.9%)		4.68 ± .02	
MODERATE:				
* Procedure #1*	.66 (.63, .69)	.7 (.7, .7)	−.031 (−.043, −.02)	−.024 (−.036, .012)
* Procedure #2*	6.5 (6.3, 6.8)	7.4 (7.1, 7.7)	.90 (.88, .91)	.87 (.85, .88)
* Procedure #3*	7.4 (7.1, 7.7)	9.0 (8.7, 9.4)	.18 (.17, .19)	.17 (.15, .18)
* Procedure #4*	5.8 (5.6, 6.0)	6.1 (5.9, 6.4)	1.06 (1.04, 1.08)	1.03 (1.01, 1.04)
* Procedure #5*	4.1 (3.9, 4.3)	4.5 (4.3, 4.7)	1.64 (1.63, 1.65)	1.61 (1.60, 1.63)
* Procedure #6*	4.7 (4.5, 4.9)	4.7 (4.5, 4.9)	.80 (.78, .82)	.78 (.76, .79)
* Procedure #7*	5.2 (4.9, 5.5)	5.2 (4.9, 5.5)	.93 (.90, .95)	.88 (.85, .90)
* Procedure #8*	5.5 (5.2, 5.8)	5.0 (4.7, 5.3)	.33 (.31, .36)	.33 (.30, .35)
* Procedure #9*	.9 (.8, .9)	.9 (.8, .9)	1.89 (1.88, 1.91)	1.86 (1.84, 1.87)
* Procedure #10*	7.1 (6.8, 7.5)	7.8 (7.4, 8.3)	.82 (.79, .84)	.78 (.76, .80)
(%) or Mean ± SEM	(7.2%)		8.18 ± .03	
P _Moderate__vs.__Mild_	<0.0001		<0.0001	
SEVERE:				
* Procedure #1*	.67 (.64, .71)	.66 (.63, .71)	−.09 (−.11, −.08)	−.088 (−.10, −.075)
* Procedure #2*	7.67 (7.43, 7.92)	8.39 (8.11, 8.68)	.63 (.62, .65)	.61 (.59, .63)
* Procedure #3*	11.09 (10.73, 11.46)	12.45 (12.02, 12.90)	−.06 (−.08, −.05)	−.093 (−.11, −.078)
* Procedure #4*	4.37 (4.22, 4.52)	4.47 (4.32, 4.63)	.85 (.84, .87)	.83 (.81, .84)
* Procedure #5*	3.68 (3.54, 3.83)	3.84 (3.69, 4.00)	1.47 (1.46, 1.48)	1.46 (1.45, 1.47)
* Procedure #6*	3.17 (3.05, 3.29)	3.21 (3.09, 3.34)	.64 (.63, .66)	.63 (.62, .65)
* Procedure #7*	1.20 (1.16, 1.25)	1.17 (1.12, 1.21)	.59 (.58, .61)	.58 (.57, .59)
* Procedure #8*	3.19 (3.06, 3.33)	3.22 (3.08, 3.36)	.23 (.21, .25)	.23 (.21, .24)
* Procedure #9*	.85 (.79, .92)	.79 (.73, .86)	1.80 (1.79, 1.82)	1.78 (1.77, 1.79)
* Procedure #10*	5.89 (5.59, 6.19)	6.26 (5.94, 6.60)	.57 (.54, .59)	.54 (.52, .57)
(%) or Mean ± SEM	(10.7%)		7.80 ± .02	
P _Severe__vs.__Mild_	<0.0001		<0.0001	
Interaction Effects:	Moderate vs Mild	Severe vs Mild	Moderate vs Mild	Severe vs Mild
* Procedure #1 x AIS*	0.208	0.077	0.884	<0.0001
* Procedure #2 x AIS*	<0.0001	<0.0001	<0.0001	<0.0001
* Procedure #3 x AIS*	0.054	<0.0001	0.014	<0.0001
* Procedure #4 x AIS*	<0.0001	<0.0001	<0.0001	<0.0001
* Procedure #5 x AIS*	<0.0001	<0.0001	<0.0001	<0.0001
* Procedure #6 x AIS*	0.002	<0.0001	<0.0001	<0.0001
* Procedure #7 x AIS*	<0.0001	<0.0001	<0.0001	<0.0001
* Procedure #8 x AIS*	<0.0001	<0.0001	<0.0001	<0.0001
* Procedure #9 x AIS*	<0.0001	<0.0001	<0.0001	<0.0001
* Procedure #10 x AIS*	<0.0001	<0.0001	<0.0001	<0.0001

∗ Adjusted for patient- and hospital-level characteristics. Procedure #1: 8659 Closure of skin and subcutaneous tissue of other sites, Procedure #2: 9604 Insertion of endotracheal tube, Procedure #3: 9671 Continuous invasive mechanical ventilation for less than 96 consecutive hours, Procedure #4: 3893 Venous catheterization (not elsewhere classified), Procedure #5: 9672 Continuous invasive mechanical ventilation for 96 consecutive hours or more, Procedure #6: 9904 Transfusion of packed cells, Procedure #7: 0131 Incision of cerebral meninges, Procedure #8: 9907 Serum transfusion (not elsewhere classified), Procedure #9: 311 Temporary tracheostomy, Procedure #10: 3891 Arterial catherization. β = slope, AIS = Abbreviated Injury Severity, CI = confidence interval, OR = odds ratio, SEM = standard error of the mean.

## 4 Discussion

In this cross-sectional study, we performed secondary analyses on hospital discharge records that corresponded to adults with TBI from the 2004 to 2014 NIS in order to identify and characterize the top 10 procedure codes and evaluate whether injury severity has an impact on prevalence, time trends, and outcomes of these specific procedures. As expected, distinct patterns of resource utilization were observed among mild, moderate, and severe TBI hospitalizations, with the most frequently identified procedure codes aimed at homeostatic stabilization as well as prevention of secondary injuries post-TBI. Specifically, results suggested that these top 10 procedure codes ranged in prevalence between 3.1% and 15.5%, with disparities in prevalence rates according to injury severity. Although the prevalence rates of these procedure codes did not exhibit a clear time trend, there were significant differences in these rates by year of admission. One potential explanation for variations in the use of procedure codes is a change in healthcare policy during the 2004 to 2014 period, including the implementation of the Affordable Care Act. In-hospital death affected 6.5% of all patients, and patients were hospitalized for an average of 6.9 days. These in-hospital outcomes differed according to injury severity, with greater severity associated with worse outcome. An inverse relationship with poor outcomes was observed between 3 out of 10 procedure codes at least in some of the injury severity groups, whereas other procedure codes were directly associated with poor outcomes irrespective of injury severity. These relationships are likely non-causal but rather can be explained by the phenomenon of confounding by indication, necessitating further investigation. It is worth noting that most patients would undergo multiple procedures during their hospitalizations, and injury severity may dictate procedure selection as well as healthcare outcomes.

The study finding that nearly equal numbers of mild, moderate, and severe TBI cases were identified based on the AIS is indicative of greater disease severity among patients who are hospitalized versus the general population whereby nearly 3-quarters had mild TBI.^[14]^ It is worth noting that AIS is among several indices of TBI severity, with similarly conducted NIS-based studies using the AIS,^[1,5]^ the “all patient refined diagnosis-related groups” (APRDRGs),^[20]^ and the “independent survival risk ratio” (SRRi),^[22]^ precluding comparisons among studies. On the other hand, recommendations for diagnosis and treatment of TBI, with an emphasis on neuroimaging, pharmacological, device-based, surgical as well as rehabilitative treatments, are largely dependent on TBI severity, as summarized in recently published literature reviews.^[3,17,23–30]^

To our knowledge, this study is the first to examine frequently utilized procedures among hospitalized US adults with TBI, using a large, nationally representative sample. Previous studies using national databases were mainly focused on trends, risk factors, comorbidities, and outcomes of TBI in various populations.^[5,10,31,32]^ Few studies involving hospitalized TBI patients from the NIS have examined utilization and outcomes of specific procedures. For instance, a recently conducted study by Chaudhry and colleagues analyzed the 2008 NIS data to evaluate incidence and risk factors for in-hospital death among patients with TBI who underwent Percutaneous Endoscopic Gastrostomy (PEG).^[22]^ Consistent with this study, researchers estimated an in-hospital mortality rate of 6%, and worse prognosis among PEG versus non-PEG patients based on age, comorbidities and high-risk status.^[22]^ Another study by Hoffman and colleagues combined 2002 to 2011 NIS databases to estimate risk factors (acquired immunodeficiency syndrome, sepsis, and cerebrospinal fluid leak) and incidence rate of meningitis (4.3%) as well as length of stay (42 days among patients with meningitis) and in-hospital mortality rates (12.8% among patients with meningitis) in patients with TBI who underwent external ventricular drain placement.^[20]^ These findings are consistent with the idea that procedure-associated complications can mitigate the risk of adverse events. In this study, we found that several invasive procedures were consistently associated with worse in-hospital outcomes, irrespective of injury severity.

The NIS database captures some but not all procedure codes that correspond to recommended procedures for moderate-to-severe TBI patients in an acute setting. According to Swanson and colleagues, emphasis is often placed on homeostatic stabilization and prevention of secondary injuries.^[33]^ For instance, neuroimaging and intraventricular catheterization can aid in the detection and monitoring of elevated intracranial pressure prior to managing elevated intracranial pressure with pharmacological treatments, controlled hyperventilation, decompressive craniectomy, and/or skull manipulation.^[33]^ Brain oxygenation may necessitate the utilization of endotracheal intubation and mechanical ventilation, and blood pressure control may be achieved using intravenous fluids and medications.^[33]^ Other procedures may be applied for tight regulation of body temperature and brain metabolism whereas neurologic surgery may be needed for the removal of foreign material or drainage of contusions and hematomas.^[33]^

Study findings should be interpreted with caution and in light of several limitations. First, we relied on an administrative database consisting of hospital discharge records which has limited information on laboratory tests and medications. Furthermore, the NIS database did not allow us to distinguish hospitalizations for isolated TBI from those resulting from polytrauma, to clearly identify a procedure code as being diagnostic versus therapeutic or to distinguish TBI-specific from procedure-specific complications. Second, data clustering as a consequence of patient re-admission to one of the participating hospitals cannot be evaluated without access to unique patient identifiers. Third, complete subject analysis was performed with potential for selection bias because of missing data. Fourth, many study variables were defined based on ICD-9 codes, potentially leading to misclassification bias, with an inability to distinguish major and minor procedures or the specific type of procedure (e.g., peripheral line vs central line). Particularly, injury severity was defined based on diagnostic codes rather than using the standard definition that combines GCS, LOC, and PTA criteria. Top 10 procedure codes had relatively low prevalence rates since we did not combine these codes to reflect specific procedure types. Fifth, residual confounding may have led to biased measures of association. Sixth, this study design does not allow for establishment of temporality or causal relationships between exposure and outcome variables. Without randomization, the observed associations between procedures and in-hospital outcomes may be partly due to confounding by indication, whereby patients with better or worse prognosis are more likely to receive specific procedures. Finally, study findings may be generalized to hospitalized patients whose characteristics may differ from those who sought outpatient care.

This exploratory and hypothesis-generating analysis identified the top 10 procedure codes applied to hospitalized adults with TBI. Knowledge of patterns of healthcare utilization can establish a baseline for the planning of future healthcare resource needs as well as the implementation and evaluation of quality improvement projects focused on optimizing therapies and follow-up of TBI patients within hospital settings. Since we evaluated procedure codes rather than types of procedures, the estimated prevalence rates were relatively low. Furthermore, prevalence rates of top 10 procedure codes varied by injury severity and were associated with distinct in-hospital outcomes. It is worth noting, however, that utilization of specific procedures may be more indicative of injury severity than poor outcome. For instance, the observed relationship between death and specific procedure codes (e.g., closure of skin and subcutaneous tissue of other sites) cannot be interpreted as being causal in nature, but rather as the outcome of confounding by indication. Future research should use longitudinal data from disease registries or insurance claims databases while combining multiple procedure codes to elucidate patterns of utilization of diagnostic and/or treatment procedures. These studies will also be helpful at elucidating which specific procedures are key to achieving better healthcare utilization outcomes, taking injury severity level, and other relevant characteristics into consideration.

## Author contributions

**Conceptualization:** Hind A Beydoun.

**Data curation:** Hind A Beydoun.

**Formal analysis:** Hind A Beydoun.

**Funding acquisition:** May A. Beydoun, Shaker M. Eid, Alan B. Zonderman.

**Methodology:** Hind A Beydoun, Catherine Butt, May A. Beydoun, Sharmin Hossain.

**Project administration:** Shaker M. Eid.

**Resources:** Shaker M. Eid, Alan B. Zonderman.

**Software:** May A. Beydoun.

**Supervision:** Shaker M. Eid, Alan B. Zonderman.

**Validation:** Hind A Beydoun, Catherine Butt, Sharmin Hossain.

**Visualization:** Hind A Beydoun.

**Writing – original draft:** Hind A Beydoun.

**Writing – review & editing:** Hind A Beydoun, Catherine Butt, May A. Beydoun, Sharmin Hossain, Shaker M. Eid, Alan B. Zonderman.

## Abbreviations

Abbreviations: AHRQ = Agency for Healthcare Research and Quality, AIS = Abbreviated Injury Severity, CAT = computerized axial tomography, CCS = Clinical Classifications Software, CDC = Centers for Disease Control and Prevention, CI = confidence interval, GCS = Glasgow Coma Scale, HCUP = Healthcare Cost and Utilization Project, LOC = loss of consciousness, NIS = Nationwide Inpatient Sample, OR = odds ratio, PEG = Percutaneous Endoscopic Gastrostomy, PTA = posttraumatic amnesia, SEM = standard error of the mean, TBI = traumatic brain injury, US = United States.
